# Supplementary material for: The interactome of intact mitochondria by cross-linking mass spectrometry provides evidence for coexisting respiratory supercomplexes
Source: Mol Cell Proteomics. 2017 Dec 8;17(2):216–32. doi: 10.1074/mcp.RA117.000470 (PMC5795388; doi:10.1074/mcp.RA117.000470)
Supplement: Supplemental Data [file supp_RA117.000470_133922_0_supp_23978_fzffwf.zip › spectra_annotation/mito_DR_spectra_annotation/376-1-5-1-9-1.pdf]

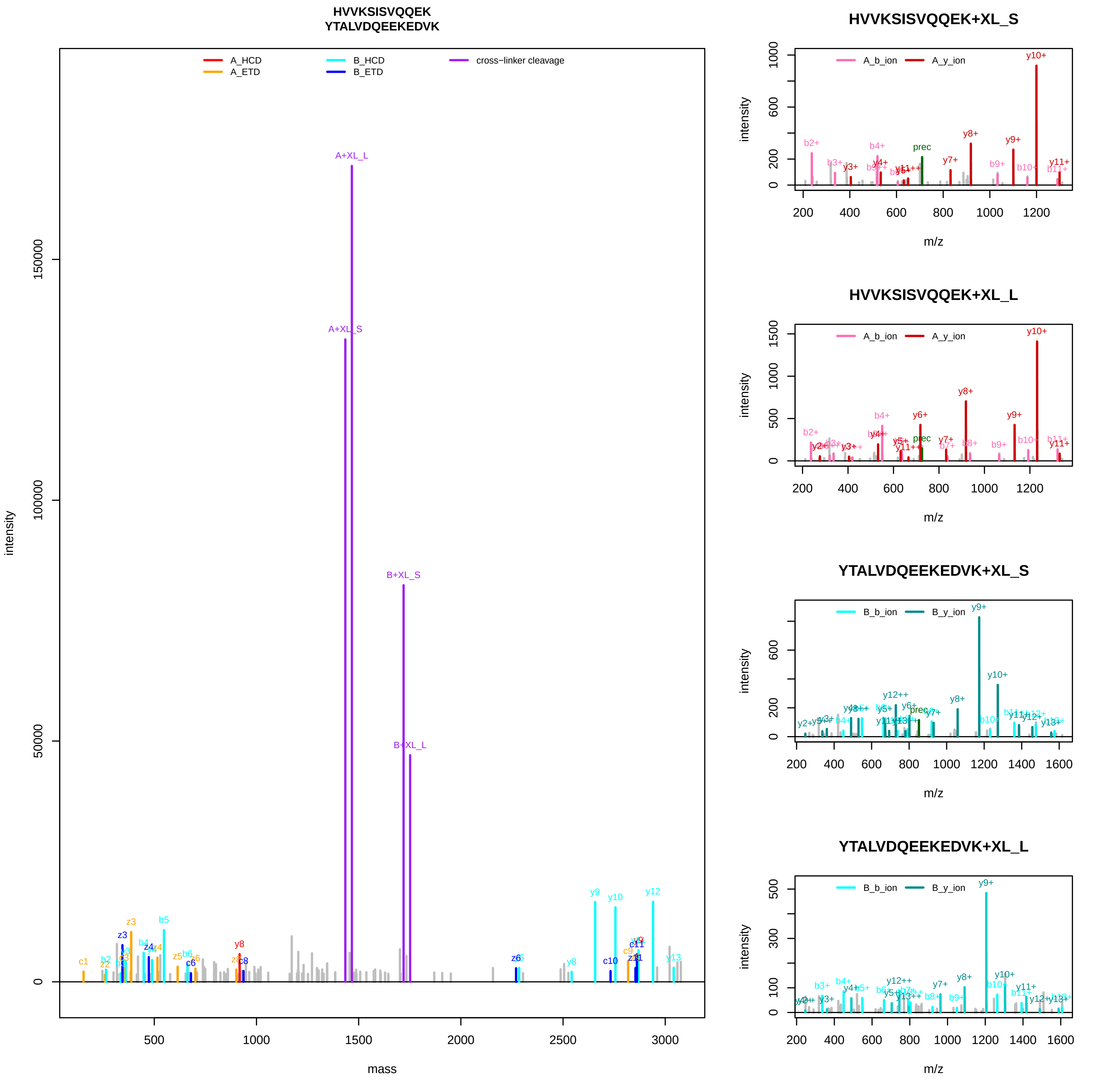
HVVKSISVQQEK
YTALVDQEEKEDVK
A_HCD
A_ETD
B_HCD
B_ETD
cross−linker cleavage
A+XL_L
A+XL_S
B+XL_S
B+XL_L
intensity
mass
0
50000
100000
150000
500
1000
1500
2000
2500
3000
c1
b2
z2
z3
b5
z3
b4
z4
y8
z5
b6
c6
c8
z6
y8
y9
y10
c10
c9
y12
c11
y13
HVVKSISVQQEK+XL_S
A_b_ion
A_y_ion
b2+
b3+
y3+
b4+
y4+
prec
y7+
y8+
y9+
y10+
b9+
b10+
y11+
b11+
m/z
HVVKSISVQQEK+XL_L
y6+
y5+
y11++
b7+
b8+
YTALVDQEEKEDVK+XL_S
B_b_ion
B_y_ion
y9+
y10+
y12++
y8+
y6+
y5+
b10+
y11+
y12+
y13+
y2+
y3+
YTALVDQEEKEDVK+XL_L
b3+
b4+
y4+
b5+
y12++
y7+
y8+
y13++
b8+
b9+
b10+
b11+
y11+
y12+
y13+
0
100
300
500
200
400
600
800
1000
1200
1400
1600
